# Journal of Neonatal Surgery Celebrates Second Anniversary

**Published:** 2014-01-01

**Authors:** Yogesh K Sarin, Bilal Mirza

**Affiliations:** 1 Department of Pediatric Surgery, Maulana Azad Medical College, New Delhi, India; 2 Department of Pediatric Surgery, The Children’s Hospital and the Institute of Child Health Lahore, Pakistan

Journal of Neonatal Surgery, the premier quarterly, peer-reviewed journal focusing on surgical conditions of the neonates, celebrated the completion of its second volume with the publication of the Sept-Dec 2013 issue. It is the first journal of its kind, serving as an international forum to disseminate the knowledge about neonatal surgery and its allied subjects. The growth and interest sparked during the first two years of publication has been remarkable. We have maintained the initial excitement and published every issue on time.

Last year, the Journal received 57 manuscripts from around the world of which 52 were accepted for publication. 

The top five manuscripts that had highest viewership in the year 2013 are listed below. We congratulate each of their authors.

1. Saifullah M, Ferdous KMN, Shahjahan M, Sayed SA. Management of Congenital Talipes Equino Varus (CTEV) by Ponseti Casting Technique in Neonates: Our Experience. . J Neonat Surg. 2013; 2: 17 (3051 views)
2. Celayir CA, Sahinoglu Z, Selcuk S, Moraliglu S, Bosnali O. Unilateral Huge Hydronephrosis Necessitating Fetal Interventions (2060 views)
3. Krishnan L. Pain Relief in neonates J Neonat Surg. 2013; 2: 19. (971 views)
4. Patel RV, Kumar H, More B. Preampullary duodenal web simulating gastric outlet obstruction. J Neonat Surg. 2013; 2: 13. (927 views)
5. Haidar AM, Gharmool BM. Extracorporeal testicular ectopia through inguinal canal: a case report. J Neonat Surg. 2013; 2: 10. (726 views)

It is very interesting to note that the maximum viewership this year was for an original article on a musculo-skeletal anomaly (read orthopedic) from none other than a center in Bangladesh. 

We once again wish to express my sincere gratitude to our reviewers for their valuable time, as well as to our authors and the Journal's Editorial Board for their most valuable support. 

On its second anniversary, we suggest that we consider launching a formal society associated with the journal. We would be looking forward to hear the views of the editorial board and all-important readership in this regard. Indexing with Pubmed is in pipeline. 

We also wish to infuse some fresh blood in the Editorial Board. We invite applications from our worthy global colleagues for the honorary positions on the Editorial Board. 

We trust our faithful readership would keep supporting us in our academic endeavor.

## Footnotes

**Source of Support:** Nil

**Conflict of Interest:** None

